# Supplementary material for: Flash drug release from nanoparticles accumulated in the targeted blood vessels facilitates the tumour treatment
Source: Nat Commun. 2022 Nov 14;13:6910. doi: 10.1038/s41467-022-34718-3 (PMC9661469; doi:10.1038/s41467-022-34718-3)
Supplement: Supplementary file 1 — Supplementary Information [file 41467_2022_34718_MOESM1_ESM.pdf]

Flash drug release from nanoparticles accumulated in the targeted blood vessels facilitates the tumour treatment

Ivan V. Zelepukin[1,2]*, Olga Yu. Griaznova[1,2], Konstantin G. Shevchenko[3,4], Andrey V. Ivanov[5], Ekaterina V. Baidyuk[3], Natalia B. Serejnikova[5], Artur B. Volovetskiy[5], Sergey M. Deyev[1,2,5]* & Andrei V. Zvyagin[1,2,5,6]*

[1] Shemyakin-Ovchinnikov Institute of Bioorganic Chemistry of the Russian Academy of Sciences, 117997 Moscow, Russia;

[2] National Research Nuclear University MEPhI (Moscow Engineering Physics Institute), 115409 Moscow, Russia;

[3] Institute of Cytology of the Russian Academy of Sciences, 194064 Saint Petersburg, Russia;

[4] Chumakov Federal Scientific Center for Research and Development of Immunobiological Drugs of the Russian Academy of Sciences, 108819 Moscow, Russia;

[5] Sechenov First Moscow State Medical University (Sechenov University), 119991 Moscow, Russia;

[6] MQ Photonics Centre, Macquarie University, 2109 Sydney, Australia;

* Corresponding author e-mails: Zelepukin I.V.: zelepukin@phystech.edu; Deyev S.M.: deyev@ibch.ru; Zvyagin A.V.: andrei.zvyagin@mq.edu.au

Supplementary Information

Supplementary Note 1. Drug release model

Drug carriers are assumed to be in capillaries and to start the release of a low molecular weight drug at the time of adherence to endothelium ($t = 0$). Perfect mixing of the drug in the vessel is assumed. We aim to model a drug transport from capillary to interstitium with subsequent internationalisation by cancer cells. Comparison of the intracellular drug concentration between several drug delivery modes is the goal of this modelling. We are solving the problem using the hybrid compartmental model[1] and the Krogh cylinder model,[2] as reported by Eikenberry in relation to the drug delivery to tumour tissue.[3] A single capillary of radius r_c and length L is considered to be in the centre of the axis of a cylindrical tissue region of radius r_t , the Krogh's cylinder radius. We assume the capillary exchanges a drug only with surrounding finite cylindrical tissue region. The condition of zero radial solute flux is imposed at the outer edge of the Krogh's cylinder. The rationale behind this assumption is that the tissue cylinder is embedded in a capillary network modelled as the array of equivalent cylinders. This capillary network nurtures whole organ,

whereas single capillary nurtures only the tissue in its vicinity, and the vicinity represents the Krogh's cylinder. We adopt the following simplifying assumptions:[4]

1. Axial diffusion is neglected in both the capillary and tissue regions.
2. Convective drug transport in the radial direction, which is driven by the hydraulic and osmotic pressure difference is neglected.
3. At the capillary-tissue interface drug is equilibrated step-wisely between the tissue and capillary, and the process is characterised by the permeability coefficient, P.
4. The interstitial and capillary tissues are homogeneous.
5. Drug absorption in the vessel is neglected.

Two drug delivery modes were considered. Free drug delivery (Case FDD): intravenous injection of free drug; and encapsulated drug delivery (Case EDD): intravenous injection of drug encapsulated in nanocontainers, with ensuing 80% ID sequestration in the lungs (fractional coefficient β = 0.8). Rapid and slow (quasi-continuous over an extended period) administration rates were compared for both delivery modes modelled by a Heaviside function, as drug administration duration period T = 3 min and 3 h, respectively. The drug dose D was equal for all delivery modes and administration rates.

First, we analysed the case of FDD using doxorubicin as a model drug. The drug quickly distributed from the systemic circulation to the body compartments at the minute-time scale via blood vessels and capillaries. Upon cessation of the drug administration, drug was cleared from tissues through capillaries, the process was characterised by the time constant α (Supplementary Table 1). In the simplest case of a one-compartment pharmacokinetic model, the drug concentration in the capillary is expressed as:

$$C_v(t) = \begin{cases} \frac{DA}{T\alpha}[1 - \exp(-\alpha t)], \; t < T \\ \frac{DA}{T\alpha}[\exp(-\alpha T) - 1]\exp(-\alpha t), \; t > T \end{cases}, \qquad (1)$$

where A – compartment 1 parameter [L^{-1}]. Drug transport from the capillary to the tissue is determined by the permeability of the capillary wall, P. In the tissue, the drug transport is governed by passive diffusion in accordance with the Fick's law. Cells absorb doxorubicin in accordance with the Michaelis-Menten law. Besides, doxorubicin quickly binds to plasma proteins, primarily, albumin with the association rate k_a and dissociate with the dissociation rate k_d, their ratio is stipulated as k_a/k_d = 3. A set of partial differential equations governing the time evolution of free (C_f) and albumin-bound (C_b) drug concentrations as well as intracellular drug concentration (C_i) are given as

$$\frac{\partial C_f}{\partial t} - D_f \nabla^2 C_f + \rho_c \zeta \mu - \rho_c \zeta \nu + k_a C_f - k_b C_b = 0 \ , \tag{2}$$

$$\frac{\partial C_b}{\partial t} - D_b \nabla^2 C_b - k_a C_f + k_b C_b = 0 \ , \tag{3}$$

$$\frac{\partial C_i}{\partial t} = \mu - \nu \ , \tag{4}$$

$$\mu = V_{\mathrm{m}} \frac{(C_f + C_b)}{C_f + C_b + K_E \phi} \ , \tag{5}$$

$$\mu = V_{\mathrm{m}} \frac{C_i}{C_i + K_I} \ . \tag{6}$$

∇^2 is the Laplace operator in cylindrical coordinates [$\nabla^2 C = \frac{1}{r}\frac{\partial}{\partial r}(r\frac{\partial C}{\partial r})$]; D_f, D_b – diffusivity of free and bound doxorubicin, respectively; μ, ν – cellular uptake, efflux rate of doxorubicin, respectively, ρ_c – cell density, ζ – scaling coefficient; V_m – maximum reaction rate; K_E, K_I – Michaelis coefficients for the extracellular and intracellular doxorubicin, respectively.

Boundary conditions are used to account for an influx of doxorubicin at the capillary wall:

$$\left.\frac{\partial C_f}{\partial r}\right|_{r=r_c} = P_F[\theta(1-\delta)C_v - C_f(r_c, t)] \tag{7}$$

$$\left.\frac{\partial C_b}{\partial r}\right|_{r=r_c} = P_B[\theta\delta C_v - C_b(r_c, t)] \ , \tag{8}$$

where P_f, P_B – permeability of the capillary to free and bound doxorubicin, respectively; θ – fraction of plasma in the blood; δ – fraction of doxorubicin bounded to plasma proteins. The tissue is bounded by a cylinder of radius r_t (Krogh cylinder[1,2] , or tumour cylinder[3]) centred at the capillary vessel. No-flux boundary conditions are used for all variables at the outer radius of the tumour cylinder.

The formalism above is applicable for the FDD case. In case of the EDD, an additional two-compartment model needs to be introduced to account for the drug delivery to the lungs compartment, which exchanges drug with the body compartment and characterised by a drug transfer rate k_{v0}. The drug exchange rate from the body to the lungs is assumed equal to that from the lungs to the body. In addition, the drug is eliminated from the body compartment at a rate k_{el}. Drug concentrations in the body (C_0) and lungs (C_v) compartments are related by the following coupled ordinary differential equations:

$$\frac{dC_0}{dt} = -k_{el}C_0 - k_{v0}C_0 + k_{v0}C_v \ , \tag{9}$$

$$\eta \frac{dC_v}{dt} = k_{v0}C_0 - k_{v0}C_v + \frac{\beta D A}{T} H(T - t) \ , \tag{10a}$$

$$\left.\frac{\partial C_v}{\partial r}\right|_{r=r_c} = -P_F[\theta(1-\delta)C_v - C_f(r_c, t)] \ , \tag{10b}$$

where η stands for the ratio of the lungs (V_l) and whole body (V_0) volumes, evaluated approximately, as 0.1,[5] β stands for the fraction of encapsulated drug delivered to the lungs. C_0 and C_v stand for doxorubicin concentrations in the body and lungs, respectively. Considering C_0 is much smaller than C_v for hours-long period which lasts until equilibrium is established, C_0 is ignored. Supplementary Equations (9) and (10a) are replaced with a factor β/η by which the right-hand sides of Supplementary Equations (7), (8) are multiplied. This holds well for EDD rapid delivery, whereas the simplified model gives an overestimate of C_b, C_i in case of EDD slow delivery. Since we aim to demonstrate the superiority of rapid EDD for drug delivery, the upper bounds of C_b, C_iin case of EDD slow delivery are deemed acceptable.

These equations were solved numerically by using a commercial software package FlexPDE 7.0 using parameters presented in Supplementary Table 1. The code used in the theoretical modelling is available at https://github.com/zelepukiny/FlaRE-delivery.[6]

Supplementary Table 1. Parameters used in numerical calculations.

Parameter	Description	Value range	Default value	Units
D_F	Free doxorubicin diffusion coefficient	0.568–3.587	0.4	$mm^2\,h^{-1}$
D_B	Bound doxorubicin diffusion coefficient	0.03276–0.2268	0.032	$mm^2\,h^{-1}$
P_F	Diffusive permeability for free doxorubicin	2.916–13.306	10	$mm\,h^{-1}$
P_B	Diffusive permeability for bound doxorubicin	0.02378–0.03242	0.032	$mm\,h^{-1}$
A	Compartment 1 parameter	15.7-130.3 E-09	7.46 E-08	mm^{-3}
α	Compartment 1 clearance rate	5.09-12.76	9.68	h^{-1}
D	Total dose of doxorubicin injected	100-285	150	mg
δ	Fraction of plasma doxorubicin bound	0.74-0.82	0.75	–
θ	Fraction of blood that is plasma	–	0.6	–
k_a	Free doxorubicin-albumin binding rate	3000–4000	3000	h^{-1}
k_d	Doxorubicin-albumin dissociation rate	–	1000	h^{-1}
V_{max}	Rate for transmembrane transport, for 10^5 cells	–	16.8	$ng\,h^{-1}$
K_E	Michaelis constant, extracellular	–	2.19 E-04	$\mu g\,mm^{-3}$
K_I	Michaelis constant, intracellular, for 10^5 cells	–	1.37	ng
ζ	Scaling factor	–	1 E-08	–

ρ_c	Density of tumour cells	0.955-15.3 E+05	1 E+06	mm^{-3}
ϕ	Tumor fraction extracellular space	0.2–0.6	0.4	–
S_{body}	Total surface area of an adult man	–	1.9	m^2
V_{lung}	Blood volume in lungs per surface area	–	271	$mL\ m^{-2}$
V_{lungs}	Blood volume in the lungs	–	0.5149	L
V_{total}	Total blood volume	–	5	L
ξ	Ratio of V_{lungs}/V_{total}	–	0.10298	–

Supplementary Fig. 1 and Fig. 1c of the main text show radial distribution of the albumin-bound doxorubicin concentration, $C_b(r)$, computed at several time points. Note that due to the fast doxorubicin-albumin association-dissociation rates, equilibrium between $C_b(r)$ and $C_f(r)$ is quickly established, so that $C_f(r)$ is 3 times lower but the functional dependence conforms that of $C_b(r)$. Interestingly, after an initial period of the drug uptake by the tumour tissue, the process is reversed, and the drug starts clearing from the tissue driven by the concentration gradient from the tissue to the capillary. The kinetics of cellular uptake of a drug is much slower and nonlinear so that the relationship between $C_b(r)$ and doxorubicin intracellular concentration $C_i(r)$ is more complex, as shown in Supplementary Fig. 2 and Fig. 1d of the main text.

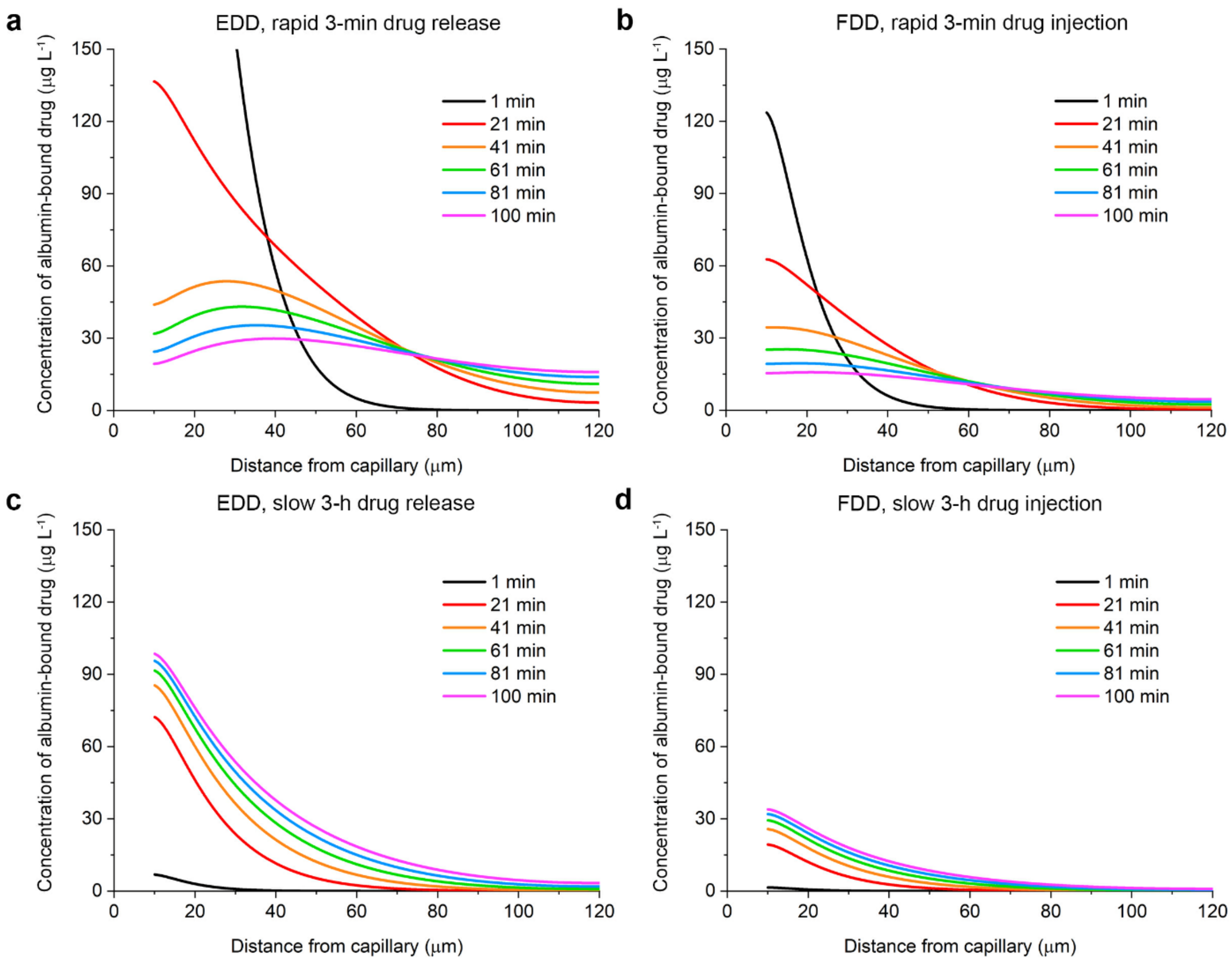

Supplementary Fig. 1. Concentration of albumin-bound doxorubicin versus the radial distance from a capillary. **a, c** Encapsulated drug delivery (EDD) mode, doxorubicin is delivered to the lungs and released during initial 3 min **(a)** or 3 h **(c)**. **b, d** Free drug delivery **(**FDD) mode, doxorubicin is administrated during initial 3 min **(b)** or 3 h **(d)**. In **a-d** kinetics at different time-points are marked with following colours: black (1 min), red (21 min), orange (41 min), green (61 min), blue (81 min), magenta (100 min).

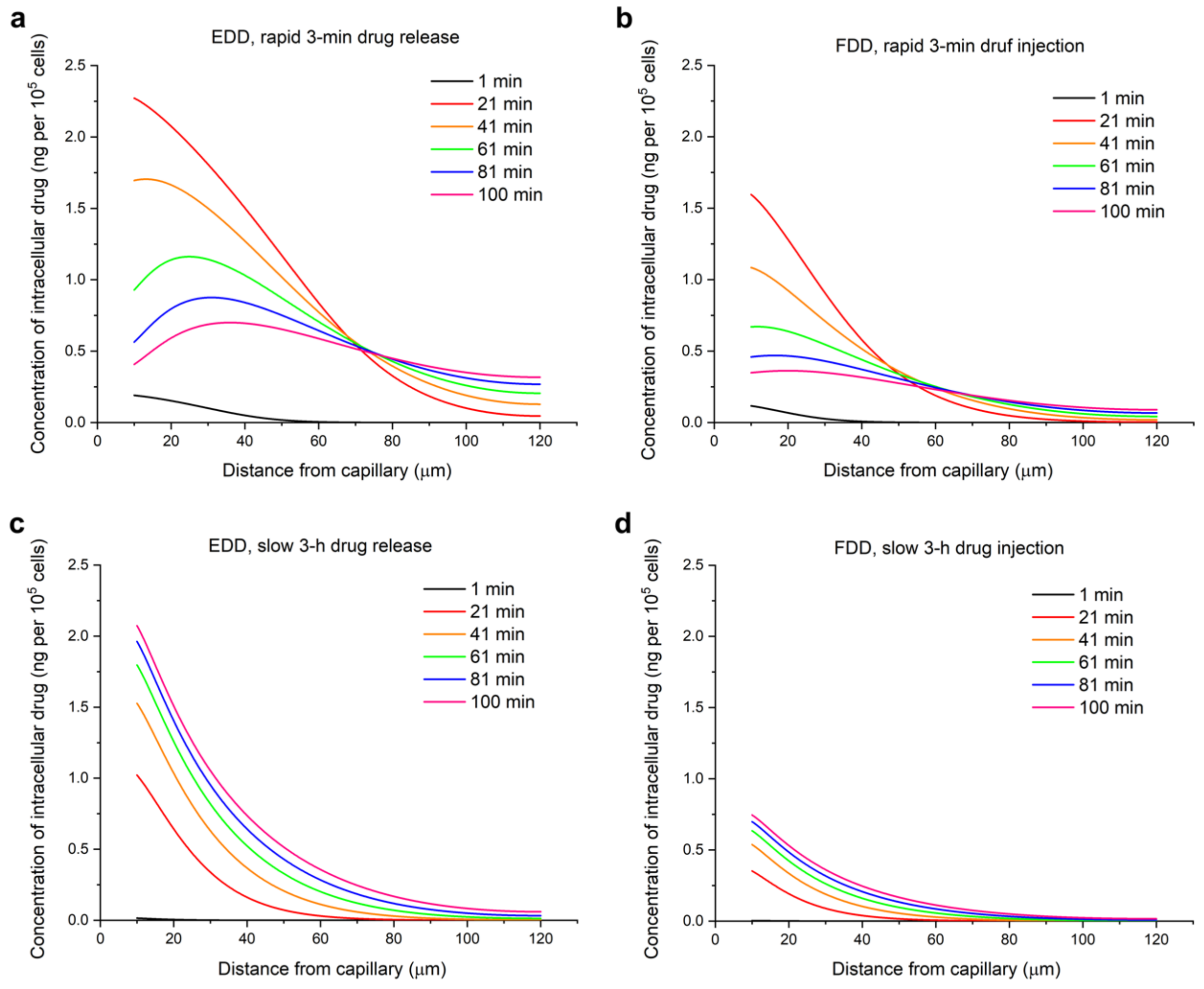

Supplementary Fig. 2. Concentration of intracellular doxorubicin versus the radial distance from a capillary. **a, c** Encapsulated drug delivery (EDD) mode, doxorubicin is delivered to the lungs and released during initial 3 min **(a)** or 3 h **(c)**. **b, d** Free drug delivery (FDD) mode, doxorubicin is administrated during initial 3 min **(b)** or 3 h **(d)**. In **a-d** kinetics at different time-points are marked with following colours: black (1 min), red (21 min), orange (41 min), green (61 min), blue (81 min), magenta (100 min).

Supplementary Table 2. Drug loading of different molecules to the MIL-101 NPs. Data are presented as mean values ± SD.

Molecule	Drug loading, w/w, %
Nile Blue	69 ± 3
Sulphorhodamine B	68 ± 5
Resazurin Sodium Salt	82 ± 1
Rhodamine B	53 ± 1
Rhodamine 590	39.9 ± 0.1
Trypan Blue	41.2 ± 0.1
Bromophenol Blue	52.7 ± 0.1
Acridine Orange	37.6 ± 0.1
Fluoresceine	60 ± 8
Vitamin B12	58 ± 1
Riboflavin Mononucleotide	60.4 ± 0.2
Indocyanine Green	79.3 ± 0.2
Doxorubicin	36.2 ± 1.4
Rhodamine 123	42 ± 3

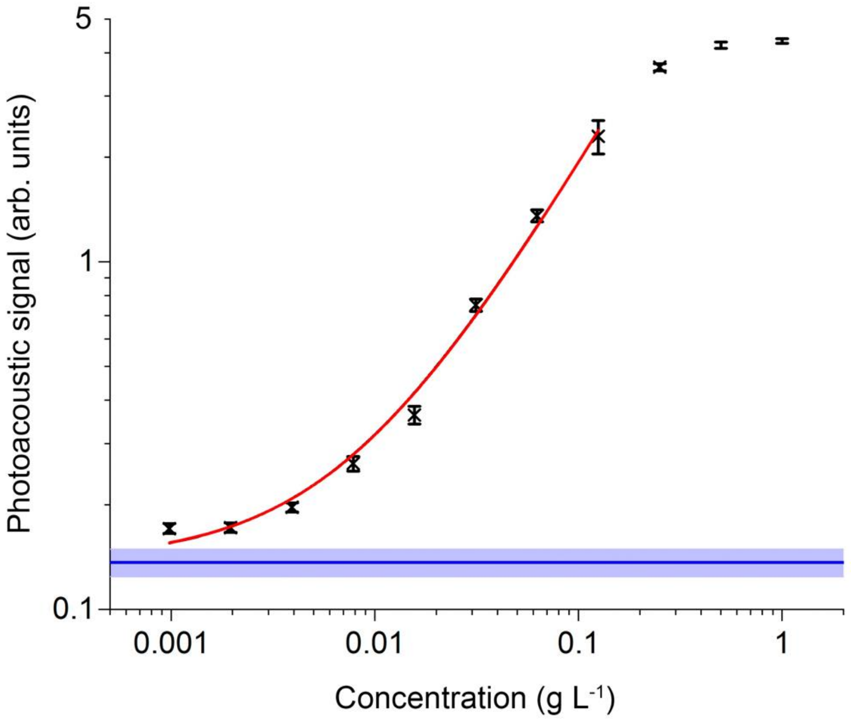

Supplementary Fig. 3. Calibration plot of the photoacoustic system for quantification of the concentration of MIL-101 NPs. n = 3 independent samples. Blue line shows photoacoustic signal from water ± 3-fold electronic noise level. Red line shows linear fitting of the data points. Linear range was 10^{-3} – 0.125 g L^{-1}. For MIL-101 NPs data are presented as mean values ± SD.

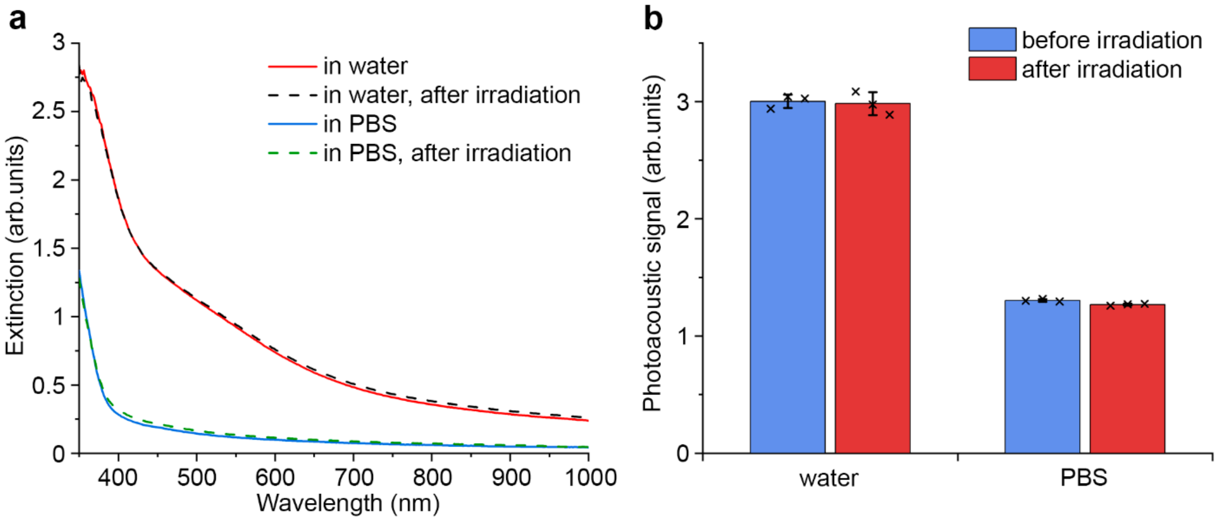

Supplementary Fig. 4. a Extinction spectra of MIL-101 NPs in water (red, black) and their degraded forms in PBS (blue, green), before (solid line) and after 15-min laser irradiation (dashed line) by photoacoustic setup. n = 1 sample. **b** Photoacoustic signals of MIL-101 NPs in water and their degraded forms in PBS before (blue) and after 15-min laser irradiation (red) by photoacoustic setup. n = 3 independent samples. Data are presented as mean values ± SD.

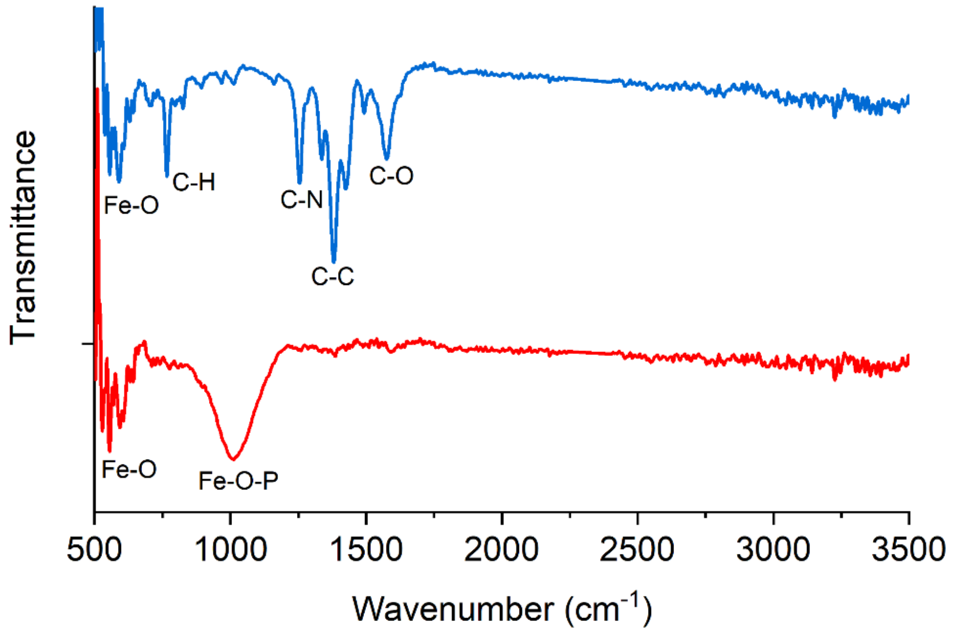

Supplementary Fig. 5. Fourier transformed infrared spectra of dry powders of MIL-101 NPs incubated overnight in water (blue) and PBS (red). n = 1 sample.

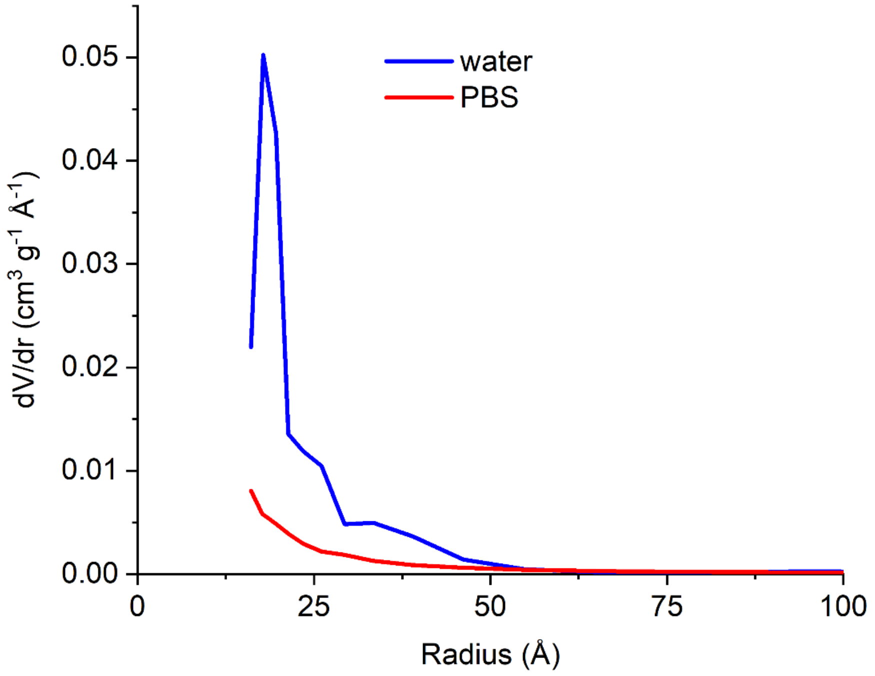

Supplementary Fig. 6. Plot of the pore volume distribution of MIL-101 NPs incubated for 24 h in water (blue) or PBS (red) versus the pore radius. n = 1 sample.

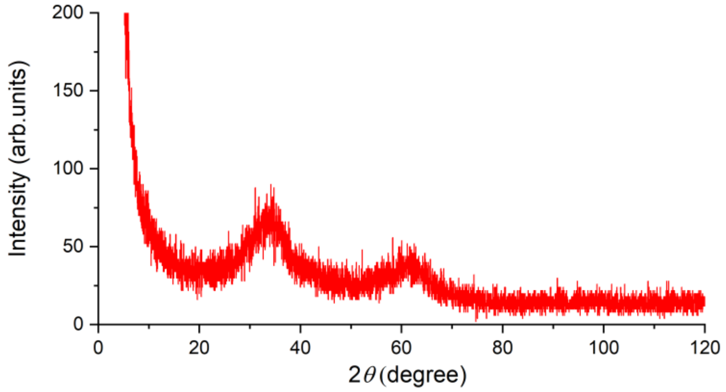

Supplementary Fig. 7. X-ray diffraction spectrum of MIL-101 NPs 24-h post incubation in PBS (pH 8.5). n = 1 sample.

Supplementary Table 3. Results of energy-dispersive X-ray spectroscopy analysis of MIL-101 NPs incubated overnight in water or in PBS. n = 3 spectra averaged.

**Water**		
Element	Weight, %	Atomic, %
C	80.58	85.37
O	12.51	9.97
Fe	2.17	0.49
N	4.45	4.06
Cl	0.30	0.11
**PBS**		
Element	Weight, %	Atomic, %
C	40.88	53.49
O	39.72	39.12
Fe	11.74	3.32
Cl	0.38	0.16
P	5.62	2.87
Na	1.32	0.90

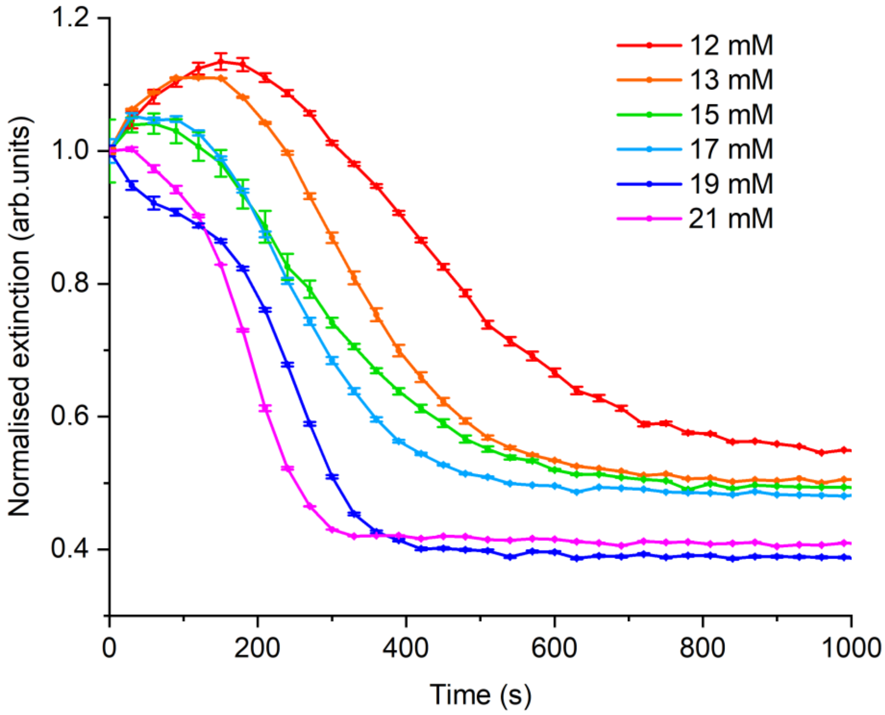

Supplementary Fig. 8. Kinetics of MIL-101 NP degradation in PBS supplemented with 50 g L^{-1} bovine serum albumin, acquired at several concentrations of phosphates in solution. Kinetics at different concentrations are marked with following colours: red (12 mM), orange (13 mM), green (15 mM), cyan (17 mM), blue (19 mM), magenta (21 mM). Extinction of the MIL-101 NPs was measured at a wavelength of 368 nm, each curve was normalised to the extinction coefficient of the corresponding colloid at t = 0 s. n = 3 independent samples. Data are presented as mean values ± SD.

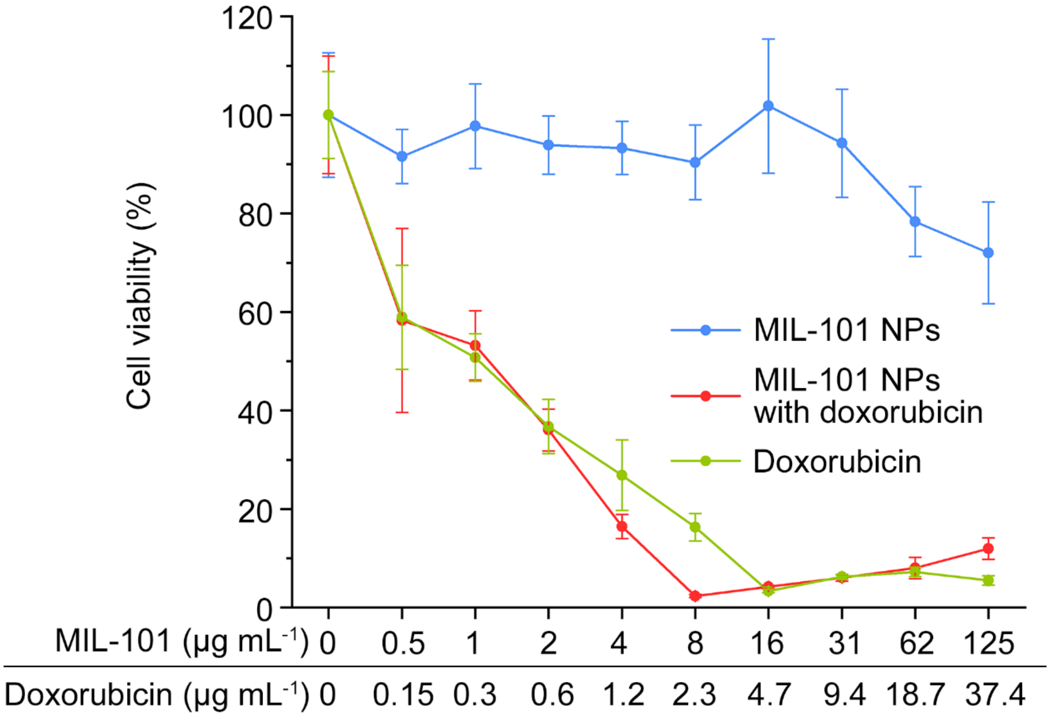

Supplementary Fig. 9. MTT cytotoxicity assay of MIL-101 NPs (blue), doxorubicin-loaded MIL-101 NPs (red) and free doxorubicin (green) in B16-F1 melanona cells. The cell viability is presented as % ratio normalised to the non-treated control cells. n = 6 samples analysed per data point. Data are presented as mean values ± SD.

Supplementary Note 2. Theoretical fitting of rhodamine 123 kinetics in the lung tissue

The kinetics of the bound drug concentration at a fixed distance from a capillary obtained in our theoretical model (c.f. Supplementary Fig. 1 sampled at the fixed distance at several time points), was fitted to an analytical function of the drug concentration, C, where the distance from a capillary, r was set to 45 μm. The analytical function for the rhodamine 123 kinetics in the lung tissue was constructed as derived by J. Crank[7] for an infinite source line in a homogeneous medium characterised by the substance desorption rate $\phi(t) = \frac{\Phi'}{l_c}$ [s^{-1} mm^{-1}]:

$$C = \frac{1}{4\pi D}\int_0^t \phi(t')\exp\left[-\frac{r^2}{4D(t-t')}\right]\frac{dt'}{t-t'} \; ,$$

where D – diffusion constant, t - time.

The numerical solution of this equation was used to fit the experimental data by the least square method using Mathematica 13.1 software. This function was used to fit the experimental data of the dye distribution. The results of the modelling are presented in Fig. 5d.

Supplementary Note 3. Histopathological evaluation of MIL-101 nanoparticle systemic toxicity as a drug delivery agent

The histopathological evaluation of MIL-101 NPs was carried out using two animal models. First, toxicity and biodistribution of unloaded MIL-101 NPs were evaluated in untreated mice. Second, systemic toxicity of doxorubicin-loaded MIL-101 NPs was evaluated using mouse melanoma lung metastasis model. Histopathological analysis was carried out by a professional histopathologist. Morphological signs of the target organs in every slide were evaluated with 0-to-3 point system: 0 – no sign; 1 – the least pronounced sign; 2 – moderately pronounced sign; 3 – the most pronounced sign.

First, we evaluated the biodistribution and systemic toxicity of MIL-101 NPs at a dose of 25 mg kg^{-1}, when administered via intravenous injection in female BALB/c mice in the tail vein. Same volume of PBS administered in mice served as the negative control. To perform biodistribution analysis, mice were sacrificed at time points ranging from 5 min to 14 days, the main organs were harvested and analysed. Histological slices were stained with eosin & Perls Prussian blue to visualise MIL-101 NPs. MIL-101 NPs were predominantly accumulated in the lungs, liver, and spleen (Supplementary Fig. 10).

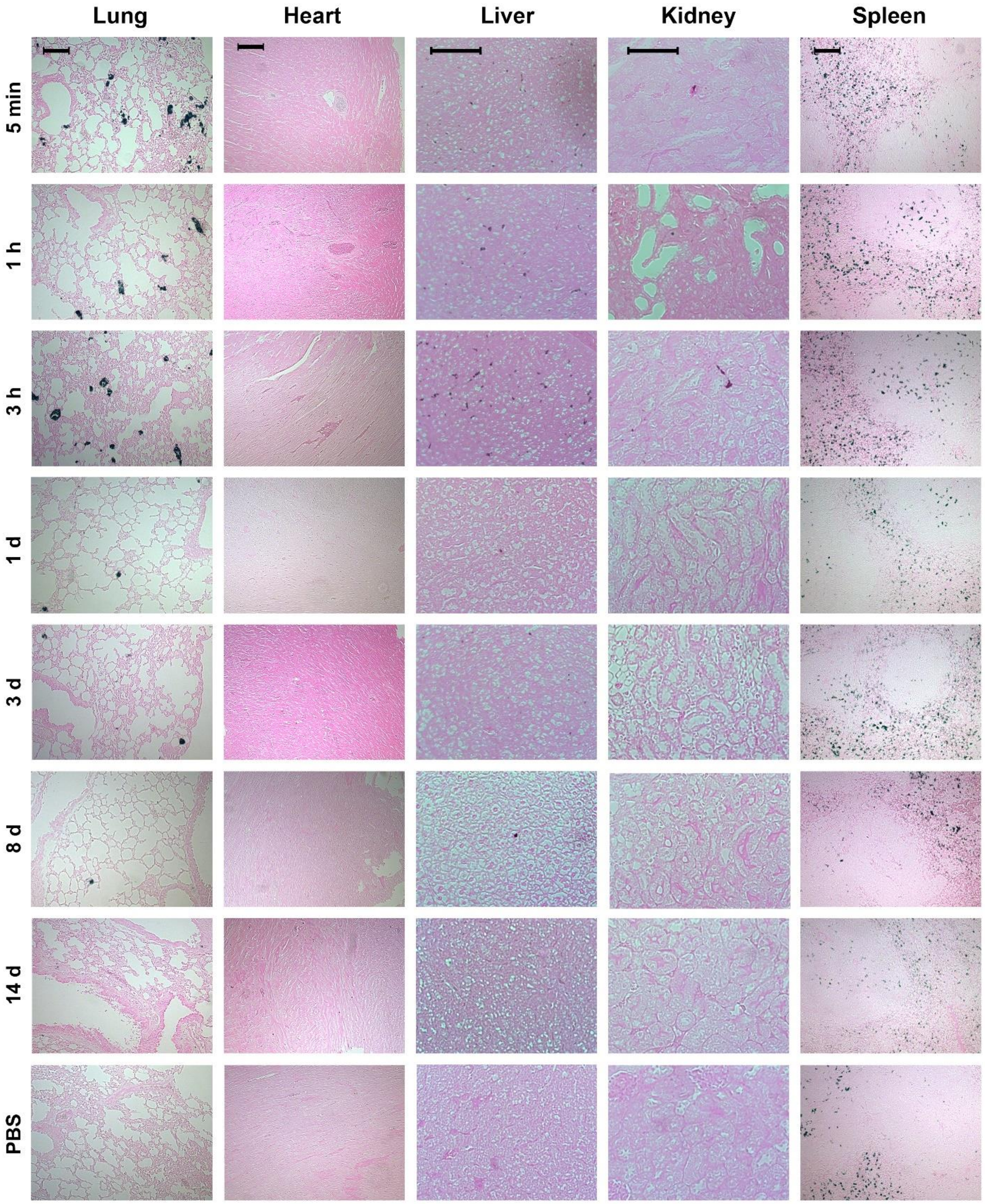

Supplementary Fig. 10. Histological images of the lung, heart, liver, spleen, and kidneys tissues at several time points after intravenous injection of MIL-101 NPs alongside with the PBS-treated control group. Tissue slices were stained with eosin and Perls Prussian blue. Scale bars = 50 μm. n = 3 mice for each time point.

To perform the toxicity studies, mice were sacrificed on days 3 and 14 post injection, and the main organs were collected and analysed. Histological slides were stained with hematoxylin & eosin. Generally, the administration of MIL-101 NPs induced negligible dystrophic changes in the structure of the analysed tissues and organs. Therefore, MIL-101 NPs can be considered safe at the tested dose. We note a slight increase in the granular dystrophy of hepatocytes, inflammatory infiltration of the stroma, a 10% increase of the average number of Kupffer cells in the liver on day 3 (Supplementary Tables 4,5). However, these abnormal changes were normalised by day 14. The structure of the other organs (the spleen, heart, kidneys, and lungs) featured no difference to that of the control for the entire duration of the experiment (Supplementary Fig. 11).

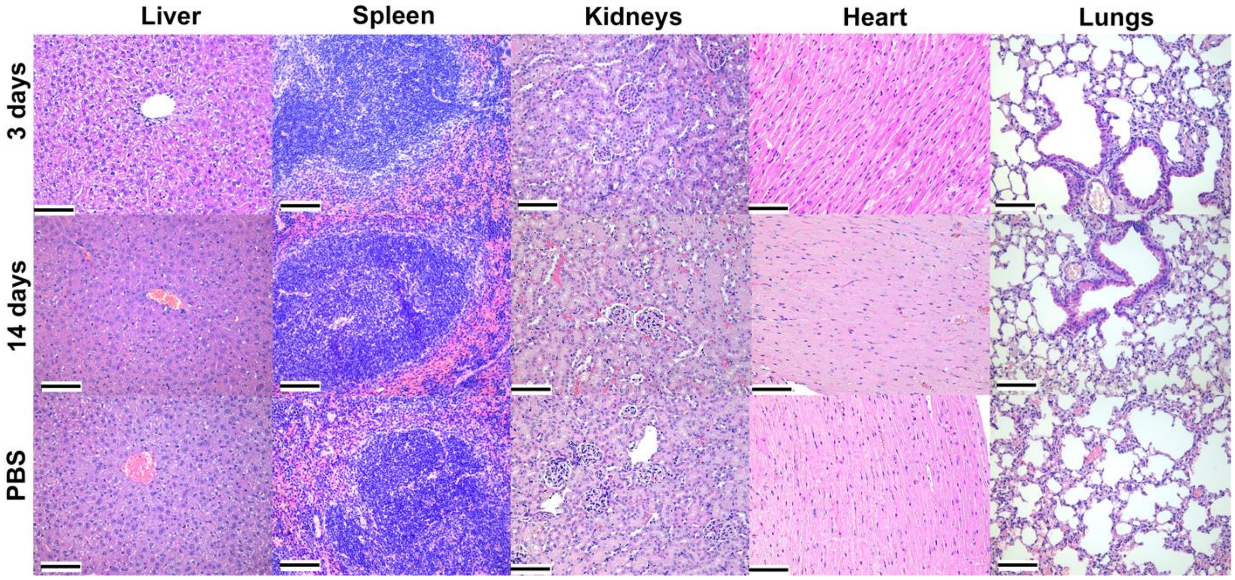

Supplementary Fig. 11. Representative images of the liver, spleen, kidneys, heart, and lung tissues stained with hematoxylin-eosin on day 3 and day 14 post intravenous injection of MIL-101 NPs. Scale bars = 100 µm. n = 3 mice per group.

Supplementary Table 4. Grading of the morphological changes of the target organs in mice on Days 3 and day 14 post injection of MIL-101 NPs. Grading scale: 0 – no sign; 1 – the least pronounced sign; 2 – moderately pronounced sign; 3 – the most pronounced sign. n = 3 mice per group for liver. n = 2 mice per group for spleen, kidneys, heart, lungs.

**Morphological signs**	**PBS**			**MIL-101 NPs Day 3**			**MIL-101 NPs Day 14**		
	Identification number of animal in the group								
**LIVER**	1	2	3	1	2	3	1	2	3
1. Granular dystrophy of hepatocytes	0	0	0	1	1	1	0	0	0
2. Fatty dystrophy of hepatocytes	0	0	0	0	0	0	0	0	0
3. Necrosis of hepatocytes	0	0	0	0	0	0	0	0	0
4. Disorganization of the microarchitecture of the hepatic lobules and tracts	0	0	0	0	0	0	0	0	0
5. Increased content of Kupffer cells	0	0	0	1	2	1	0	0	0
6. Inflammatory infiltration of the stroma	0	1	0	1	1	1	0	0	1
7. Stromal sclerosis	0	0	0	0	0	0	0	0	0
8. Enhanced hyperemia of the central veins and the sinusoids	0	0	0	0	0	0	0	0	0
9. Enhanced hyperemia of the vessels of the hepatic triads	0	0	0	0	0	0	0	0	0
10. Vasculitis	0	0	0	0	0	0	0	0	0
**SPLEEN**	1	2	3	1	2	3	1	2	3
1. Hyperplasia of lymphoid follicles	0	0	-	0	0	-	0	0	-
2. Plasmatization of peripheral follicle areas	0	0	-	0	0	-	0	0	-

3. Increased germinal centers of lymphoid follicles	0	0	-	0	0	-	0	0	-
4. Enhanced hyperemia of the red pulp	0	0	-	0	0	-	0	0	-
5. Increased content of megakaryocytes	0	0	-	0	0	-	0	0	-
6. Increased content of siderophages	0	0	-	0	0	-	0	0	-
**KIDNEYS**	1	2	3	1	2	3	1	2	3
1. Granular dystrophy of convoluted tubule epithelium	0	0	-	0	0	-	0	0	-
2. Necrosis of convoluted tubule epithelium	0	0	-	0	0	-	0	0	-
3. Dystrophy of straight tubule epithelium	0	0	-	0	0	-	0	0	-
4. Inflammatory cell infiltration of the cortex and medulla of the kidney	0	0	-	0	0	-	0	0	-
5. Hyperemia of the glomerular capillaries	0	0	-	0	0	-	0	0	-
6. Hyperemia of the cortex and medulla of the kidney	0	0	-	0	0	-	0	0	-
7. Vasculitis	0	0	-	0	0	-	0	0	-
**HEART**	1	2	3	1	2	3	1	2	3
1. Dystrophy of cardiomyocytes (muscle fibers)	0	0	-	0	0	-	0	0	-
2. Necrosis of cardiomyocytes	0	0	-	0	0	-	0	0	-
3. Increased content of cells in the perimysium	0	0	-	0	0	-	0	0	-

4. Myocardial fibrosis	0	0	-	0	0	-	0	0	-
5. Hemorrhages in the myocardium	0	0	-	0	0	-	0	0	-
6. Hyperemia	0	0	-	0	0	-	0	0	-
**LUNGS**	1	2	3	1	2	3	1	2	3
1. Atelectasis (collapse of the alveolar spaces)	0	0	-	0	0	-	0	0	-
2. Distelectasis (incomplete collapse of the alveolar spaces)	0	1	-	1	0	-	1	0	-
3. Bronchial spasm	0	0	-	0	0	-	0	0	-
4. Emphysema (enlargement of the alveolar spaces)	0	0	-	0	0	-	0	0	-
5. Hyperemia of interalveolar and peribronchial vessels	0	0	-	0	0	-	0	0	-
6. Edema (transudate in the alveoli)	0	0	-	0	0	-	0	0	-
7. Alveolar hemorrhages (red blood cells in the alveoli)	0	0	-	0	0	-	0	0	-
8. Perivascular and peribronchial inflammatory infiltrates	1	0	-	0	1	-	1	0	-
9. Desquamation of alveolar epithelium	0	0	-	0	0	-	0	0	-
10. Vasculitis	0	0	-	0	0	-	0	0	-
11. Vascular thrombosis	0	0	-	0	0	-	0	0	-
12. Hyperplasia of lymphoid follicles	0	0	-	0	0	-	0	0	-

Then we evaluated the systemic toxicity effects in C57Bl/6 mice with B16-F1 melanoma lung metastasis models. Female mice were administered via the tail vein with 10^5 B16-F1 cells. In 1 week after tumour cell administration, we observed numerous melanoma micrometastases in lungs, which started extravasation from blood vessels to the surrounding tissue (Supplementary Fig. 13).

For analysis of treatment toxicity, doxorubicin-loaded MIL-101 NPs dosed 10 mg kg^{-1} and equal dose of free doxorubicin were administrated on days 1,3, and 5 post tumor inoculation. The animals were sacrificed on day 11 post injection, and the main organs were collected. Next histological slides were stained with hematoxylin & eosin, imaged, and analysed.

The inoculation of B16-F1 cells led to pronounced changes in the liver and lungs compared to the control. For instance, the content of Kupffer cells in the liver was increased by 36% and deemed significant (Supplementary Table 5). Foci of atelectasis and distelectasis in the lungs were noticeable and expected, as well as inflammatory infiltration and multiple small pulmonary metastases (Supplementary Tables 6,7). The metastases comprised large polygonal honeycomb-shaped cells with deposits of brown pigment (melanin) in the cytoplasm, mitoses were numerous (Supplementary Fig. 12).

Injection of doxorubicin as a therapeutic agent produced partially a beneficial effect. A significant decrease by 38% of the average number of Kupffer cells was noted in the liver. Number and size of the metastases reduced by Day 11 in the lungs (Supplementary Table 7). However, doxorubicin had some toxic effects such as emphysema, hyperemia, hemorrhages, and bronchial spasm in some areas of the lungs. The number of megakaryocytes in the spleen also increased (Supplementary Fig. 14, Supplementary Table 6).

The administration of doxorubicin-loaded MIL-101 NPs showed improved results of the metastasis treatment as compared to these of free doxorubicin administration. Besides, treatment with MIL-101 NPs appeared less toxic. Number and size of the lung metastases were diminished in comparison to doxorubicin-treated group. Number of Kupffer cells significantly decreased in liver and was comparable to the number of cells in healthy mice (Supplementary Table 5). A slight detrimental effect of the MIL-101 NPs was associated with an increase of the number of siderophages in the spleen (Supplementary Fig. 14). The structure of the other organs remained within normal limits (Supplementary Tables 6,7). Thus, we consider that injection of MIL-101 NPs in the chosen dose was safe.

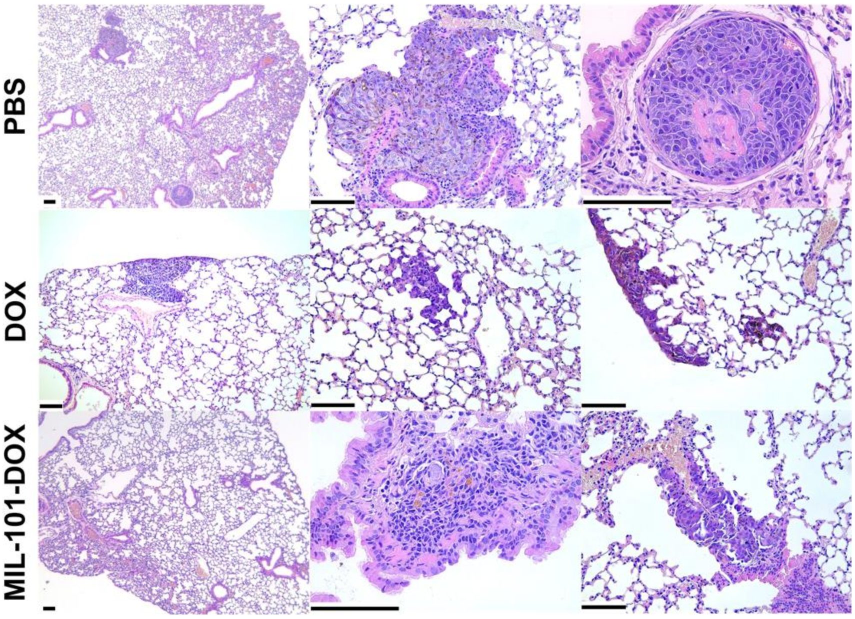

Supplementary Fig. 12. Histology images stained by hematoxylin-eosin, indicating metastases in the lungs of mice after the injection of PBS, doxorubicin (DOX) and doxorubicin-loaded MIL-101 NPs (MIL-101-DOX). Scale bars = 100 μm. n = 3 mice per group.

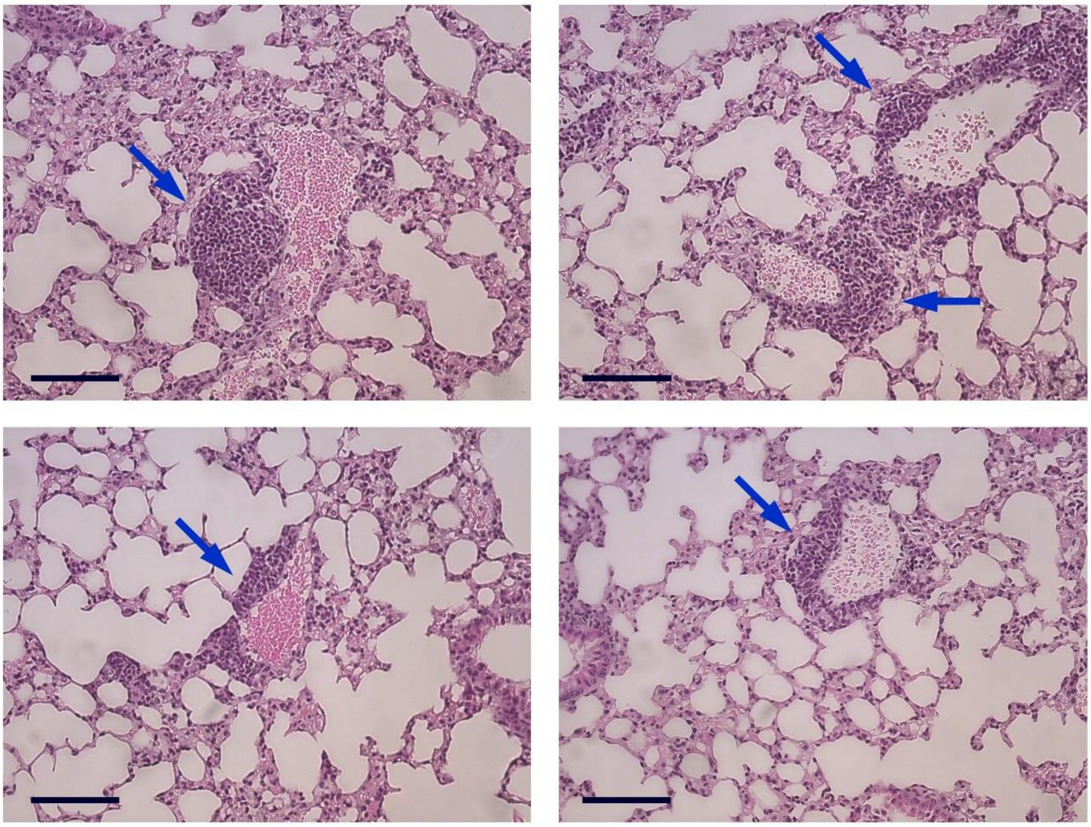

Supplementary Fig. 13. Representative histological images, showing extravasation of B16-F1 melanoma (blue arrows) from blood vessel to surrounding tissue. Scale bars = 100 μm. n = 3 mice.

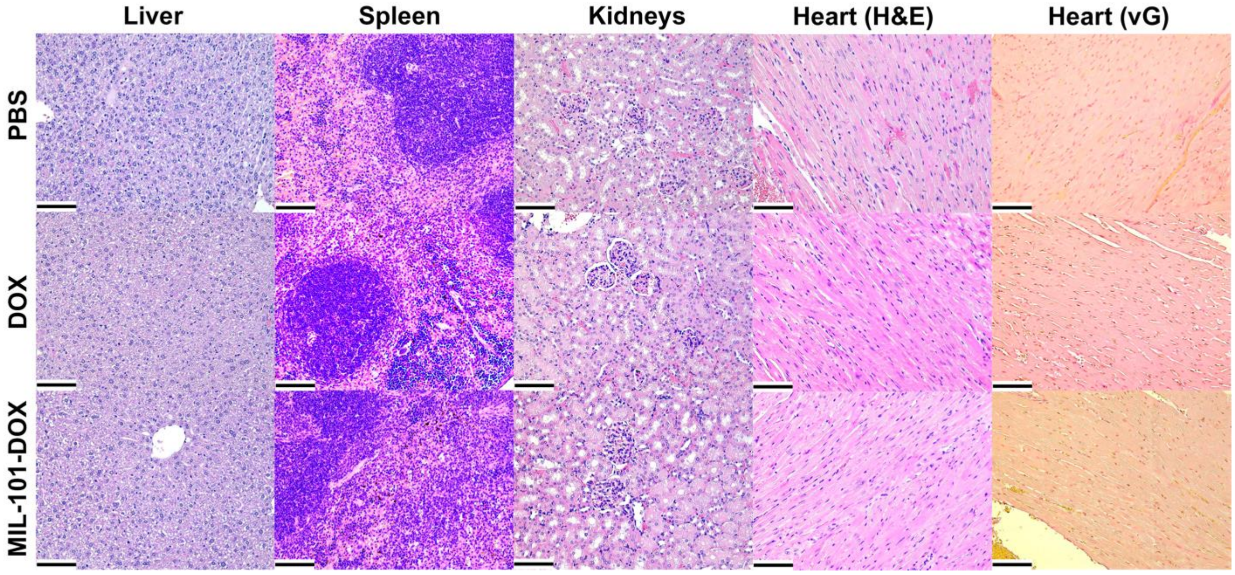

Supplementary Fig. 14. Histology images stained by hematoxylin-eosin (H&E) and by Van Gieson (vG) indicating morphological changes in the liver, spleen, kidneys, and the heart of mice after injection of PBS, doxorubicin (DOX) and doxorubicin-loaded MIL-101 NPs (MIL-101-DOX). Scale bars = 100 μm. n = 3 mice per group.

Supplementary Table 5. Morphometry of number of Kupffer cells in the liver under normal conditions and at several time points post formulation injections. n = 3 mice per group. Data are presented as mean values ± SD. Values were calculated in 10 fields of the microscope at 200× magnification for each animal in the group. One-way ANOVA with Tukey post-hoc test was used for statistical analysis. P values are shown in comparison to control (PBS) group.

<table>
<tr><th colspan="3">Healthy mice</th><th colspan="3">Tumour-bearing mice</th></tr>
<tr><td rowspan="2">Control (PBS)</td><td colspan="2">MIL-101 NPs</td><td>PBS</td><td>DOX</td><td>MIL-101-DOX</td></tr>
<tr><td>Day 3</td><td>Day 14</td><td>Day 11</td><td>Day 11</td><td>Day 11</td></tr>
<tr><td>204 ± 5</td><td>223 ± 10
P = 0.019</td><td>197 ± 10
P = 0.23</td><td>278 ± 15
P = 0.006</td><td>173 ± 10
P = 0.003</td><td>159 ± 2
P < 0.001</td></tr>
</table>

Supplementary Table 6. Grading of the morphological changes in the target organs of mice after injection of PBS, doxorubicin (DOX) and doxorubicin-loaded MIL-101 NPs (MIL-101-DOX). Grading scale: 0 – no sign; 1 – the least pronounced sign; 2 – moderately pronounced sign; 3 – the most pronounced sign. n = 3 mice for heart histology, n = 1 mice for liver, spleen, and kidneys.

Morphological signs	**PBS**			**DOX**			**MIL-101-DOX**		
	Identification number of animal in the group								
**LIVER**	1	2	3	1	2	3	1	2	3
1. Granular dystrophy of hepatocytes	0	-	-	0	-	-	0	-	-
2. Fatty dystrophy of hepatocytes	0	-	-	0	-	-	0	-	-
3. Necrosis of hepatocytes	0	-	-	0	-	-	0	-	-
4. Disorganization of the microarchitecture of the hepatic lobules and tracts	0	-	-	0	-	-	0	-	-
5. Increased content of Kupffer cells	2	-	-	0	-	-	0	-	-
6. Inflammatory infiltration of the stroma	1	-	-	1	-	-	1	-	-
7. Stromal sclerosis	0	-	-	0	-	-	0	-	-
8. Hyperemia of the central veins and the sinusoids	0	-	-	0	-	-	0	-	-
9. Hyperemia of the vessels of the hepatic triads	0	-	-	0	-	-	0	-	-
10. Vasculitis	0	-	-	0	-	-	0	-	-
**SPLEEN**	1	2	3	1	2	3	1	2	3
1. Hyperplasia of lymphoid follicles	0	-	-	0	-	-	0	-	-
2. Plasmatization of peripheral follicle areas	0	-	-	0	-	-	0	-	-
3. Increased germinal centers of lymphoid follicles	0	-	-	0	-	-	0	-	-

4. Hyperemia of the red pulp	0	-	-	0	-	-	0	-	-
5. Increased content of megakaryocytes	0	-	-	2	-	-	0	-	-
6. Increased content of siderophages	0	-	-	0	-	-	2	-	-
**KIDNEYS**	1	2	3	1	2	3	1	2	3
1. Granular dystrophy of convoluted tubule epithelium	0	-	-	0	-	-	0	-	-
2. Necrosis of convoluted tubule epithelium	0	-	-	0	-	-	0	-	-
3. Dystrophy of straight tubule epithelium	0	-	-	0	-	-	0	-	-
4. Inflammatory cell infiltration of the cortex and medulla of the kidney	0	-	-	0	-	-	0	-	-
5. Hyperemia of the glomerular capillaries	0	-	-	0	-	-	0	-	-
6. Hyperemia of the cortex and medulla of the kidney	0	-	-	0	-	-	0	-	-
7. Vasculitis	0	-	-	0	-	-	0	-	-
**HEART**	1	2	3	1	2	3	1	2	3
1. Dystrophy of cardiomyocytes (muscle fibers)	0	0	0	0	0	0	0	0	0
2. Necrosis of cardiomyocytes	0	0	0	0	0	0	0	0	0
3. Increased content of cells in the perimysium	0	0	0	0	0	0	0	0	0
4. Myocardial fibrosis	0	0	0	0	0	0	0	0	0
5. Hemorrhages in the myocardium	0	0	0	0	0	0	0	0	0
6. Hyperemia	0	0	0	0	0	0	0	0	0

Supplementary Table 7. Grading of the morphological changes in the lungs of mice after injection of PBS, doxorubicin (DOX) and doxorubicin-loaded MIL-101 NPs (MIL-101-DOX). Grading scale: 0 – no sign; 1 – the least pronounced sign; 2 – moderately pronounced sign; 3 – the most pronounced sign. n = 5 mice per group.

**Morphological signs**	**PBS**					**DOX**					**MIL-101-DOX**				
	Identification number of animal in the group														
**LUNGS**	1	2	3	4	5	1	2	3	4	5	1	2	3	4	5
1. Atelectasis (collapse of the alveolar spaces)	0	0	1	2	1	0	0	0	1	0	1	0	0	1	0
2. Distelectasis (incomplete collapse of the alveolar spaces)	1	2	2	2	2	2	2	1	1	1	2	1	1	1	2
3. Bronchial spasm	0	0	0	0	0	1	1	0	1	0	0	0	1	0	0
4. Emphysema (enlargement of the alveolar spaces)	0	0	0	0	1	1	1	1	2	1	1	0	1	1	0
5. Hyperemia of interalveolar and peribronchial vessels	1	0	0	0	0	0	2	2	0	0	0	1	0	0	1
6. Edema (transudate in the alveoli)	0	0	0	0	0	0	0	0	0	0	0	0	0	0	0
7. Alveolar hemorrhages (red blood cells in the alveoli)	0	0	0	0	0	1	1	1	0	0	0	1	0	0	0
8. Perivascular and peribronchial inflammatory infiltrates	1	1	1	1	1	1	0	0	1	0	1	1	0	0	0

9. Desquamation of alveolar epithelium	0	0	0	0	0	0	0	0	0	0	0	0	0	0	0
10. Vasculitis	0	0	0	0	0	0	0	0	0	0	0	0	0	0	0
11. Vascular thrombosis	0	0	0	0	0	0	0	0	0	0	0	0	0	0	0
12. Hyperplasia of lymphoid follicles	1	1	1	1	1	0	0	0	0	0	0	0	0	0	0
13. Lung metastases	0	1	3	1	2	2	1	1	0	1	1	0	1	1	0

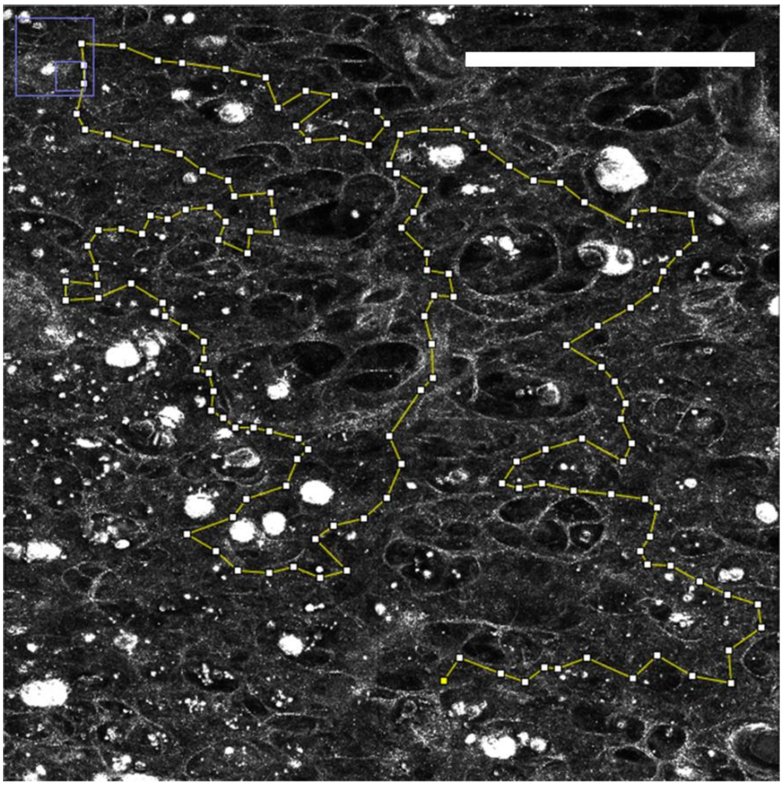

Supplementary Fig. 15. Example of the confocal image processing for measuring the fluorescence intensity of the lung tissue after the injection of rhodamine 123 loaded MIL-101 NPs. The intensity was determined by ImageJ 1.8.0 software as the mean over the line in the tissue away from the air alveolar borders and identified blood vessels (see yellow line as example). Scale bar = 250 μm.

Supplementary References

1. El-Kareh, A. W. & Secomb, T. W. A mathematical model for comparison of bolus injection, continuous infusion, and liposomal delivery of doxorubicin to tumor cells. *Neoplasia* **2,** 325–338 (2000).
2. Krogh, A. The number and distribution of capillaries in muscles with calculations of the oxygen pressure head necessary for supplying the tissue. *J. Physiol.* **52,** 409–415 (1919).
3. Eikenberry, S. A tumor cord model for doxorubicin delivery and dose optimization in solid tumors. *Theor. Biol. Medical Model.* **6,** 16 (2009).
4. Kirkpatrick, J. P., Brizel, D. M. & Dewhirst, M. W. A Mathematical Model of Tumor Oxygen and Glucose Mass Transport and Metabolism with Complex Reaction Kinetics. *Radiat. Res.* **159,** 336–344 (2003).
5. Haber, S., Clark, A. & Tawhai, M. Blood flow in capillaries of the human lung. *J. Biomech. Eng.* **135,** 101006–101011 (2013).
6. Zelepukin, I.V. et al. Flash drug release from nanoparticles accumulated in the targeted blood vessels facilitates the tumour treatment. *Zenodo*, 10.5281/zenodo.7247445 (2022).
7. Crank, J. The Mathematics of Diffusion. *Oxford university press* (1979).
